# Enhancement Mechanism of the Dynamic Strength of Concrete Based on the Energy Principle

**DOI:** 10.3390/ma11081274

**Published:** 2018-07-24

**Authors:** Jie Ren, Faning Dang, Huan Wang, Yi Xue, Jianyin Fang

**Affiliations:** 1 School of Civil Engineering and Architecture, Xi’an University of Technology, Xi’an 710048, China; ren_jie1989@163.com (J.R.); wang_huan1992@163.com (H.W.); xueyi@xaut.edu.cn (Y.X.); fjylxr@163.com (J.F.); 2 State Key Laboratory of Eco-hydraulics in Northwest Arid Region, Xi’an University of Technology, Xi’an 710048, China; 3 Shaanxi Key Laboratory of Loess Mechanics and Engineering, Xi’an University of Technology, Xi’an 710048, China

**Keywords:** concrete, dynamic strength, energy conversion, dynamic Brazilian disc test, split Hopkinson pressure bar

## Abstract

This paper analyzes the relationship between the rates of change of elastic strain energy, the strength during the concrete failure process, and proposes that the increased dynamic strength of concrete was caused by the hysteresis effect of energy release—according to the basic principle of energy conversion. Dynamic Brazilian disc tests were carried out on concrete specimens, with diameter of 100 mm, by using the split Hopkinson pressure bar. Test results were obtained through using a gas gun, with an impact pressure of 0.15 MPa, 0.20 MPa and 0.25 MPa, respectively. The dynamic failure process of concrete is then reproduced by numerical calculation methods. Finally, the energy characteristics during the concrete failure process at different strain rates are studied, and the enhancement mechanism of the dynamic strength of concrete is verified. The results showed that the dynamic tensile strength of concrete increased by 9.79% when the strain rate increased by 61% from 60.25 s^−1^; and when the strain rate increased by 92.8% from 60.25 s^−1^, the dynamic tensile strength of the concrete rose by 46.28%. The rates of change of both input energy and dissipated energy meet at the peak stress of the material. The increases in rates of change for the two kinds of energy were not synchronized, so excess input energy could be stored as concrete strength increased. As a result, the extra energy stored after peak stress led to a higher degree of concrete fragmentation and greater kinetic energy of the fragment. These results offer research directions for improving the dynamic strength of concrete.

## 1. Introduction

As the most commonly used building material, concrete is employed widely in various civil and military buildings, such as high-rise residential buildings, bridges, tunnels, hydropower stations, and military bunkers. In the design of important buildings, stability under dynamic loads, such as earthquakes, impacts, and explosions, must be considered. Since the dynamic mechanical properties and the static mechanical properties of concrete materials are quite different, it is necessary to study the mechanical properties of concrete under dynamic loads.

As early as 1917, Abrams [1] found that the dynamic strength of concrete materials increased with strain rate. Since then, a century has passed and many researchers have conducted a large number of experiments using dynamic loading equipment [2,3,4] to study the effect of strain rate on the dynamic strength of concrete.

Guo et al. [5] studied the dynamic improvement factor of concrete with different strengths, and discovered that the dynamic improvement factor of high-strength concrete was lower than low-strength concrete. Li et al. [6] studied the influence of specimen shape and size on the static and dynamic compressive strength of concrete. They proposed the relationship between the compressive strengths of cubic and cylindric specimens, and obtained an empirical formula for correlating the strengths of specimens of different sizes. Wang et al. [7] studied the stress‒strain relationship and the failure mode for roller compacted concrete under dynamic loads, and proposed an empirical formula for the improvement factor of dynamic compressive strength of roller compacted concrete. Levi-Hevroni et al. [8] improved the large-diameter split Hopkinson pressure bar (SHPB) equipment, conducted a direct tensile test on concrete, and carried out a numerical simulation on the test. Golewski and Sadowski [9,10] presented a comprehensive study of the cracking processes of concrete specimens containing fly ash and the fracture toughness of concrete was determined in terms of the critical stress intensity factors.

Clearly, recent research has focused mainly on the response of concrete under dynamic loads, while only a few studies have been carried out on the enhancement mechanism of the strength of concrete under dynamic loads.

Qi et al. [11] believe that the sensitively of concrete strength to strain rate may be regarded as the result of competition between the coexisting thermally activated and macro-viscous mechanisms. Rossi [12] holds the view that when the strain rate of concrete is less than 1 s^−1^, the enhancement of its dynamic strength is caused by the Stefan effect. Hao et al. [13] studied the effects of the end friction confinement and inertia on the compressive properties of concrete under impact loads in the SHPB test, then proposed an empirical formula. Zhang et al. [14] suggested that the increased dynamic strength of concrete resulted from the radial constraint aroused by the effect of inertia.

Due to the limitations of test equipment, tests and theoretical methods have not progressed far enough in revealing the enhancement mechanism of the strength of concrete under dynamic loads. With the development of computer technology, the combination of numerical methods and tests will better reveal the dynamic mechanical properties of concrete.

Lee et al. [15] concluded that, under loads with different rates, the increase in the dynamic compressive strength of concrete was affected by the acceleration and density of axial strain as well as the specimen shape. Zhou et al. [16] stated that the increased dynamic strength of concrete materials is caused by radial inertia and end friction effects. When the strain rate is low, enhancement of the dynamic strength of concrete is mainly caused by the end friction effect. When the strain rate is high, the radial inertia effect plays an increasingly dominant role. Hao et al. [17,18] reached the same conclusion, and considered that the larger the size of the specimen in the SHPB test, the more obvious the radial inertial effect will be. They also put forward the method of removing the inertial effect in tests. Cotsovos and Pavlovic [19] concluded that the increase in dynamic tensile strength was a structural response rather than a strain rate effect after they conducted a numerical simulation of the uniaxial tensile strength of concrete. Huang et al. [20] concluded that the lateral inertia confinement and the meso-structures were the main mechanisms for the dynamic strength enhancements.

The findings of previous studies revealed that, although a large number of researchers believe that the increased dynamic strength of concrete is caused by radial inertia and end friction effects, it seems that there is a lack of understanding with regard to the mechanisms of both radial inertia and friction. Therefore, a unified understanding has not yet been reached with regard to the enhancement mechanism of the dynamic strength of concrete.

This paper analyzes the energy conversion process of concrete under dynamic loads from the micro perspective, and proposes a mechanism for the increased dynamic strength of concrete based on the energy principle. Dynamic Brazilian disc tests were carried out on concrete using the SHPB, and the test results were compared with the proposed enhancement mechanism of dynamic strength. Numerical methods were applied to reproduce the dynamic Brazilian disc test of concrete and the energy conversion process during the dynamic failure of concrete specimens was analyzed to verify the proposed enhancement mechanism of the dynamic strength of concrete.

## 2. Enhancement Mechanism of the Dynamic Strength of Concrete

The failure process for concrete and rock materials is a thermodynamic process of energy conversion and transmission, which satisfies the law of conservation of energy. Furthermore, energy conversion is the essential feature of the physical processes of matter, reflecting the inherent attributes of materials [21,22]. Therefore, studying the dynamic strength of concrete from the perspective of energy can better reveal the failure mechanism.

During the loading process, the energy input by the system is converted into various types of energy, including elastic strain energy, plastic strain energy, fracture energy, kinetic energy, thermal energy, and radiant energy [21]. In order to better study the enhancement mechanism of the dynamic strength of concrete, the process of energy conversion is simplified and smaller energies, such as radiation energy and plastic strain energy, are ignored.

### 2.1. The Changing Process of Elastic Strain Energy during Loading

Stress waves input and transmit energy by changing the positions of atoms under dynamic loads. This generates forces between atoms and allows forces to be stored in the form of elastic strain potential energy [23]. When the elastic strain energy reaches the limit of energy storage, the atomic bonds break and the elastic strain energy is released and converted into irreversible fracture energy. In this process, the external input energy is fully converted into elastic strain energy, and the fracture energy is also converted from the elastic strain energy that has accumulated to the limit. It can therefore be considered that, during the dynamic loading of concrete, the elastic strain energy is the determining factor of concrete strength.

Concrete is a non-homogeneous material made by mortar, aggregate, and interface. Due to internal weak parts such as initial microcracks, the limits of elastic strain energy storage vary from one part to another. The weak parts may be damaged even in the early stages of loading. The energy of the concrete at the macro level is the sum of that of each microscopic part. Therefore, at the macro level, starting from the initial stage of loading, the total energy increases due to the input energy converting into the elastic strain energy of the concrete specimen. At the same time, as a result of the failure of the weak parts, elastic strain energy is transformed into irreversible energies. The entire loading process of the specimen is accompanied by constant absorption and release of elastic strain energy.

The loading of concrete is essentially a process of absorption and release of elastic strain energy. According to the law of conservation of energy, the elastic strain energy of a concrete specimen at different times can be expressed as

(1) $$e_{e}(t{)=e}_{0}(t)-e_{c}(t)$$

where $e_{e}(t)$ is the elastic strain energy of the concrete specimen at time *t*; $e_{0}(t)$ is the input energy of the loading system at time *t*; and $e_{c}(t)$ is the dissipated energy (including plastic strain energy, fracture energy, kinetic energy, heat energy, radiant energy, etc.) of the specimen at time *t*.

By Equation (1), taking the derivative of time *t*, it is shown that

(2) $$e_{e}^{\prime }(t)=e_{0}^{\prime }(t)-e_{c}^{\prime }(t)$$

where $e_{e}^{\prime }(t)$ is the variation of the elastic strain energy of the specimen per unit time; $e_{0}^{\prime }(t)$ is the variation of the input energy of the specimen per unit time; and $e_{c}^{\prime }(t)$ is the variation of the dissipated energy of the specimen per unit time.

**Figure 1:**
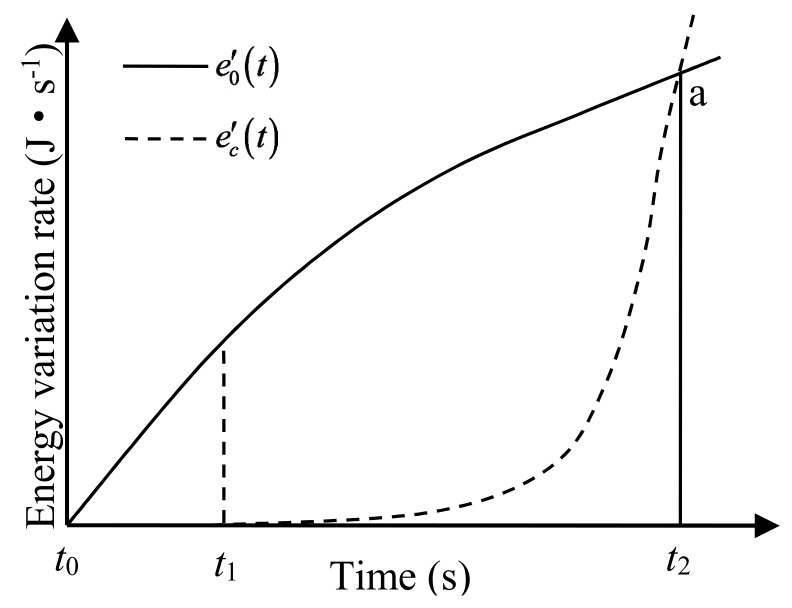
Energy variation rate of concrete at different times.

As the input energy increases, inner areas of the concrete with higher internal strength gradually fail, leading to a growing increase in dissipated energy. There is always a moment when $e_{0}^{\prime }(t)=e_{c}^{\prime }(t)$ $\left(e_{e}^{\prime }(t)=0\right)$ are valid. At this time, the elastic strain energy of the concrete reaches its storage limit. After this moment, the increment of dissipated energy exceeds that of the input energy. It can be seen from Equation (2) that the increment of elastic strain energy at that time is negative, the release of the energy dominates, and the concrete gradually breaks down. This process is shown in Figure 1.

Different states of energy change correspond to different stress-strain states. Here, *t*_0_‒*t*_1_ denotes the elastic stage, when the elastic strain energy is completely stored in the specimen. Furthermore, *t*_1_‒*t*_2_ denotes the development stage of microcracks when the elastic strain energy is partially converted into dissipated energy and is stored at the same time. At *t*_2_, the conversion rate of dissipated energy is equal to that of the input energy. At this moment, the increment of elastic strain energy becomes 0, indicating that the specimen has reached the ultimate stress state, and the stress of the material is the strength of the concrete. After *t*_2_, the stress is reduced and the specimen gradually breaks down. In Figure 1, the area enclosed by curve $e_{0}^{\prime }\left(t\right)$ and straight lines *t*_0_‒*t*_2_ and *t*_2_‒a represent the total input energy of the loading system when the concrete specimen reaches its peak stress. The area enclosed by curves $e_{0}^{\prime }\left(t\right)$ and $e_{c}^{\prime }\left(t\right)$ represent the elastic strain energy storage limit of the concrete specimen, and the area enclosed by curve $e_{c}^{\prime }\left(t\right)$ and straight lines *t*_1_–*t*_2_ and *t*_2_–a represent dissipated energy when the concrete specimen reaches its peak stress.

### 2.2. Enhancement Mechanism of the Dynamic Strength of Concrete

The macroscopic strength of concrete is determined by the rates of change of input energy and dissipated energy. The energy input rate of the loading system under impact loading increases as impact velocity rises, as does the storage rate of elastic strain energy. The higher the elastic strain energy, the greater the energy stored in the crack tip of the concrete specimen, and the higher the propagation speed of cracks. Since the dissipated energy before peak stress is mainly provided by the energy consumed during crack propagation in the concrete, it can be expressed as

(3) $$e_{c}(t)=\nu t\cdot G_{f}$$

The conversion rate of dissipated energy can be obtained by Equation (3) taking the derivative of time *t*

(4) $$e_{c}^{\prime }(t)=\nu \cdot G_{f}$$

where *v* is the propagation speed of the crack; and $G_{f}$ is the energy consumed per unit area of crack propagation. It can be seen that the higher the loading rate, the faster the crack propagation speed, and the higher the rate of change of dissipated energy.

The rates of change of input energy and dissipated energy increase with loading rate. However, there is a limit [24] for crack propagation speed under the dynamic loading effect and it does not accelerate infinitely with increased loading rate. Furthermore, the crack propagation rate contains a growing process, and the process lags behind the input of energy. Therefore, it can be considered that when the loading rate exceeds a certain threshold value, although the crack area has reached the state with ultimate stress under quasi-static or low-rate loading effect, and the elastic strain energy stored internally can cause further damage to the specimen, the increment of dissipated energy is still less than that of the input energy at that time because of hysteresis of the crack propagation rate. Hence, excess input energy continues to be stored in the specimen in the form of elastic strain energy, thus enhancing the dynamic strength of concrete.

It can be considered that the improvement of the dynamic strength of concrete is caused by hysteresis of the crack propagation rate. In terms of energy, this phenomenon can be referred to as the “hysteresis effect of energy release”.

## 3. Dynamic Brazilian Disc Test

Given that the tensile strength of concrete is far less than its compressive strength, failure of concrete caused by uniaxial compression is also called radial tensile failure. Therefore, investigating its tensile strength will help to reveal the mechanism of strength enhancement. Concrete is a multi-phase composite material. For this reason, large-diameter equipment must be used for dynamic loading. However, due to the constraints of the existing test equipment, the use of dynamic direct tensile tests in research is less common, and instead the dynamic Brazilian disc test methods have been more widely adopted.

The two main types of impact splitting test are the dynamic Brazilian disc test [25,26], and the semi-disc splitting test [27,28]. The dynamic Brazilian disc test requires neither the introduction of cracks nor any grooving into the specimen, thus making it relatively convenient. However, this method easily leads to stress concentration in the contact parts of the incident bars and the transmission bars, resulting in advanced failure. Therefore, Wang et al. [29] improved the method by introducing two parallel planes into the disc as the loading surfaces, thus solving the problem.

### 3.1. Testing Principles

**Figure 2:**
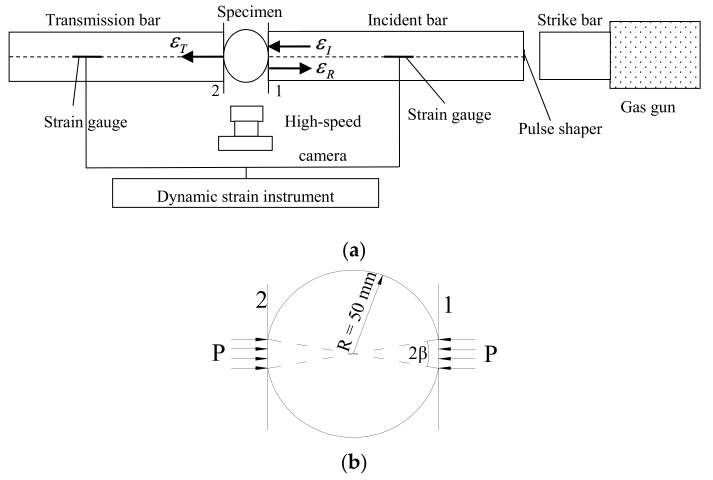
Split Hopkinson pressure bar (SHPB) arrangement used for the Brazilian tests. (**a**) Schematic of Split Hopkinson pressure bar (SHPB) test; (**b**) force deformation of the specimen.

The dynamic Brazilian disc test is based on the Brazilian disc test [30] under quasi-static load. When the SHPB device is used for the test, the loading method and forced deformation of the specimen during loading are as shown in Figure 2 [31].

According to the one-dimensional elastic wave propagation theory [32], displacements $u_{1}$ and $u_{2}$ of the two contacting end surfaces between the specimen and the elastic bars can be expressed as

(5) $$u_{1}=\int_{0}^{t}C_{0}\varepsilon_{1}dt$$

(6) $$u_{2}=\int_{0}^{t}C_{0}\varepsilon_{2}dt$$

where *C*_0_ is the wave speed of the incident and transmission bars, and $\varepsilon_{1}$ and $\varepsilon_{2}$ are the strains for the two fracture surfaces, respectively.

In the impact process, when the incident waves reach the contacting end surface 1 between the incident bar and the specimen, reflected waves are generated. Thus, the strain $\varepsilon_{1}$ at the end surface 1 includes two parts, namely the strain $\varepsilon_{I}$ of the incident waves and the strain $\varepsilon_{R}$ of the reflected waves, while $\varepsilon_{1}=\varepsilon_{I}-\varepsilon_{R}$. Putting these numbers into Equation (5), it can be shown that

(7) $$u_{1}=\int_{0}^{t}C_{0}\varepsilon_{1}dt=\int_{0}^{t}C_{0}\left(\varepsilon_{I}-\varepsilon_{R}\right)dt$$

The displacement of the end surface 2 is related only to the transmitted waves. It can be stated as

(8) $$u_{2}=\int_{0}^{t}C_{0}\varepsilon_{2}dt=\int_{0}^{t}C_{0}\varepsilon_{T}dt$$

The displacement difference between the two end surfaces is the displacement *u_s_* in loading

(9) $$u_{s}=u_{1}-u_{2}=\int_{0}^{t}C_{0}\left(\varepsilon_{I}-\varepsilon_{R}-\varepsilon_{T}\right)dt$$

According to the one-dimensional elastic wave theory and with time taken into consideration, the loads of the end surfaces 1 and 2 are, respectively

(10) $$P_{1}\left(t\right)=EA_{e}\left[\varepsilon_{I}\left(t\right)+\varepsilon_{R}\left(t\right)\right]$$

(11) $$P_{2}\left(t\right)=EA_{e}\varepsilon_{T}\left(t\right)$$

where *E* is the elastic modulus of incident bar and transmission bar; and *A_e_* is the cross-sectional area of the incident bar and transmission bar.

The average loading force *P*(*t*) at both ends of the specimen is

(12) $$P\left(t\right)=\frac{P_{1}\left(t\right)+P_{2}\left(t\right)}{2}=EA_{e}\frac{\varepsilon_{I}\left(t\right)+\varepsilon_{R}\left(t\right)+\varepsilon_{T}\left(t\right)}{2}$$

When half-sine waves are used for loading, both ends of the specimen can reach an equilibrium in force. Therefore, an assumption of equilibrium can be introduced

(13) $$\varepsilon_{I}+\varepsilon_{R}=\varepsilon_{T}$$

Putting Equation (13) into Equations (9) and (12), the following equation can be obtained

(14) $$u_{s}=\int_{0}^{t}C_{0}\left(\varepsilon_{I}-\varepsilon_{R}-\varepsilon_{T}\right)dt=-2\int_{0}^{t}C_{0}\varepsilon_{R}dt$$

(15) $$P\left(t\right)=EA_{e}\varepsilon_{T}\left(t\right)$$

The above is the general equation of loading and displacement for using the SHPB device for the dynamic Brazilian disc test. By calculating with the maximum load *P*(*t*)_max_, combining the characteristics of the flattened Brazilian disc, and setting the loading angle *β* as 10°, the indirect tensile strength [33] can be obtained

(16) $$\sigma_{t}=0.96\frac{2P{\left(t\right)}_{\max}}{\pi DL}$$

where *D* is the specimen diameter and *L* is the specimen thickness.

### 3.2. Energy Calculating Method

In the dynamic Brazilian disc test, by taking the derivatives of time *t* with respect to the incident energy $e_{I}$, the reflected energy $e_{R}$, and the transmitted energy $e_{T}$, the increments of these energies of the specimen at different times can be obtained as

(17) $$e_{I}^{\prime }\left(t\right)=A_{e}EC_{0}\varepsilon_{I}^{2}$$

(18) $$e_{R}^{\prime }\left(t\right)=A_{e}EC_{0}\varepsilon_{R}^{2}$$

(19) $$e_{T}^{\prime }\left(t\right)=A_{e}EC_{0}\varepsilon_{T}^{2}$$

According to the law of the energy conservation, it can be seen that the energy input into the specimen at different times is

(20) $$\begin{array}{ll} e_{0}^{\prime }\left(t\right) & =e_{I}^{\prime }\left(t\right)-e_{R}^{\prime }\left(t\right)-e_{T}^{\prime }\left(t\right)\\ & =A_{e}EC_{0}\varepsilon_{I}^{2}-A_{e}EC_{0}\varepsilon_{R}^{2}-A_{e}EC_{0}\varepsilon_{T}^{2}\\ & =A_{e}EC_{0}\left(\varepsilon_{I}^{2}-\varepsilon_{R}^{2}-\varepsilon_{T}^{2}\right) \end{array}$$

The elastic strain energy is partially converted into dissipated energy when the energy input into the specimen fully converts to elastic strain energy. Putting Equation (20) into Equation (2), the conversion rate of the elastic strain energy of the specimen at different times is

(21) $$e_{e}^{\prime }\left(t\right)=A_{e}EC_{0}\left(\varepsilon_{I}^{2}-\varepsilon_{R}^{2}-\varepsilon_{T}^{2}\right)-e_{c}^{\prime }\left(t\right)$$

When $e_{e}^{\prime }\left(t\right)=0$ is valid, the specimen reaches its peak stress.

### 3.3. Specimen Preparation and Experimental Arrangement

**Figure 3:**
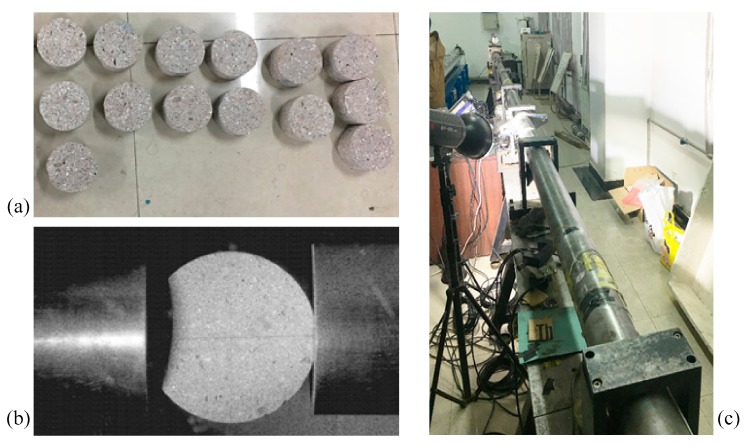
(**a**) Concrete specimen; (**b**) specimen setup; (**c**) incident bar and transmission bar.

The SHPB device and the concrete specimen are shown in Figure 3. The research object was the concrete, which had an aggregate volume fraction of 37% and a diameter varying from 5 mm to 20 mm. Clean natural river sand, with the fineness modulus of 2.4 and tap water, were employed. The water/cement ratio was 0.4 and the sand/cement ratio was 2.04 by weight.

First, the mixtures of concrete were stirred uniformly and poured into the cubic molds slowly. Second, the specimens were covered with a plastic membrane, then the molds were removed after 24 h. Third, the specimens were placed in the standard curing room at a temperature of 20 °C and humidity of 95% for 28 days. Fourth, the cubic specimens were drilled into cylindrical specimens with a diameter of 100 mm by using a drilling machine. Fifth, the concrete specimens were cut into thicknesses of 5 mm, and the end faces were made smooth and parallel by using a grinding machine. Finally, two parallel platforms with a loading angle 2β=20° at each end were ground. The specimens are shown in Figure 3a. The specimen was placed between the incident bar and the transmission bar, and the platform of the specimen was in contact with the end face of the bar (as shown in Figure 3b).

In the experimental test, the impact velocity of the strike bar is given by releasing pressure stored in the gas gun. Change in strain rate can be achieved by varying the pressure of the gas gun. The striker bar is launched to impinge against the pulse charge and generate an incident wave, which propagates towards the incident bar and the specimen. The incident wave, filtered by the pulse shaper, is sequentially transmitted to the incident bar, the specimen, and the transmission bar. Due to the impedance mismatch between the specimen and the incident bar, part of the incident wave is transmitted through the specimen into the transmission bar (transmitted wave), and part of it is reflected back towards the impact end of the incident bar (reflected wave). The incident wave and the reflected wave are extracted from the strain gauge attached to the incident bar. The transmitted wave is extracted from the strain gauge attached to the transmission bar. The dynamic mechanical properties of concrete can be calculated using the above three waves.

The 6.0 m incident bar and the 4.0 m transmission bar were made of steel (as shown in Figure 3c). Elastic modulus was *E_b_* = 210 GPa, density *ρ* = 7850 kg/m^3^, longitudinal wave velocity *C_b_* = 5172 m/s, Poisson’s ratio *ν* = 0.25, and the diameter of the bars was 100 mm. With red copper T2 as a pulse shaper, the R-value of the strain gauge was R = 1000 Ω, and the sensitivity coefficient was *k* = 1.90.

## 4. Analysis of Test Results

### 4.1. Relationship between Dynamic Tensile Strength and Strain Rate

**Figure 4:**
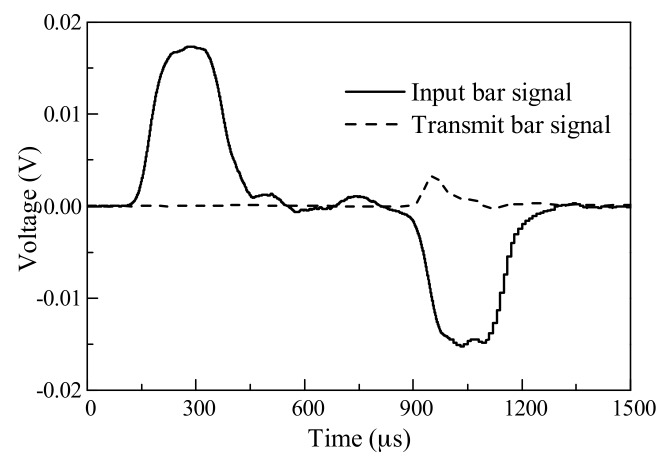
Typical wave forms.

**Figure 5:**
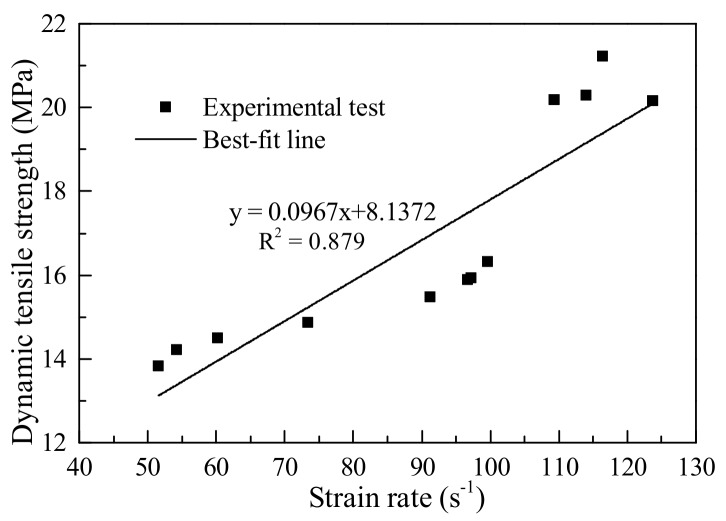
Dynamic tensile strength of concrete at different strain rates.

Figure 4 shows the waveform pulses of typical concrete specimens with 0.1 μs data acquisition frequency. Using Equations (14)–(16) to calculate the force, displacement, and average strain rate of the concrete subjected to different impact velocities, specific results were obtained as shown in Table 1. The maximum difference between the dynamic tensile strength of different concrete specimens subjected to the same impact velocity was no more than 1.06 MPa, indicating a fair regularity of test results. Figure 5 shows the relationship between the dynamic tensile strength and the strain rate of the concrete. When the strain rate was increased by 61% from 60.25 s^−1^, the dynamic tensile strength rose by 9.79%; and when the strain rate was increased by 92.8% from 60.25 s^−1^, the dynamic tensile strength of the concrete rose by about 46.28%. It can be seen that, as the strain rate increased, the dynamic tensile strength increment of concrete also increased, and the higher the strain rate, the higher the dynamic tensile strength of concrete. Lu et al. [34] obtained the same rules in the experimental study. They conducted dynamic tests with strain rates ranging from 10^−^^4^ to 10^2^. The experimental data showed that the tensile strength increased apparently with strain-rate when the strain-rate was above a critical value of around 10^0^‒10^1^ s^−^^1^.

### 4.2. Force-Displacement Relationship

**Figure 6:**
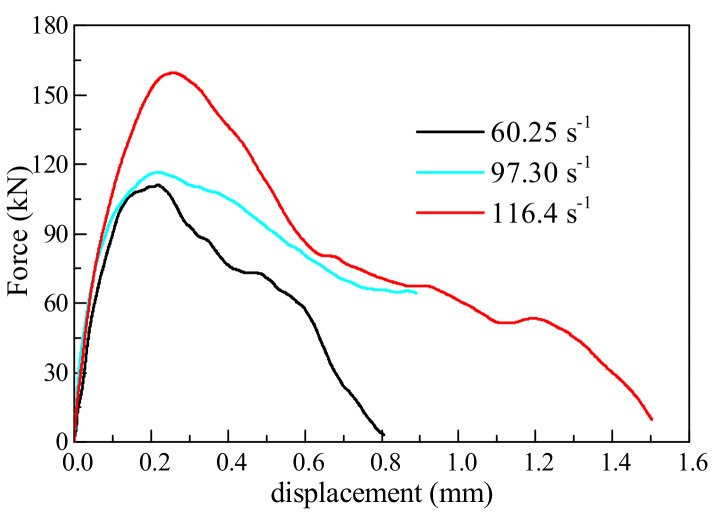
Force-displacement curves for concrete at different strain rates.

Figure 6 shows the force-displacement curves of the No. 2 specimen, No. 7 specimen, and No. 11 specimen at strain rates of 60.25 s^−1^, 97.30 s^−1^, and 116.4 s^−1^, respectively. At different strain rates, the elastic moduli of the concrete were essentially the same. At the elastic strain stage, the energy storage capacity of concrete per unit of strain remains unchanged—this is the inherent property of concrete that does not change with the increase in strain rate. The higher the strain rate, the higher the percent conversion of input energy. When concrete reaches peak strain under static conditions, the specimens do not fail in time to release energy, so the input energy is stored in the concrete; resulting in expansion of the area enclosed by the stress and strain on the coordinate diagram, and increases in peak strain and peak stress. The above phenomena are in agreement with the proposed enhancement mechanism of the dynamic strength of concrete.

### 4.3. Failure Process

**Figure 7:**
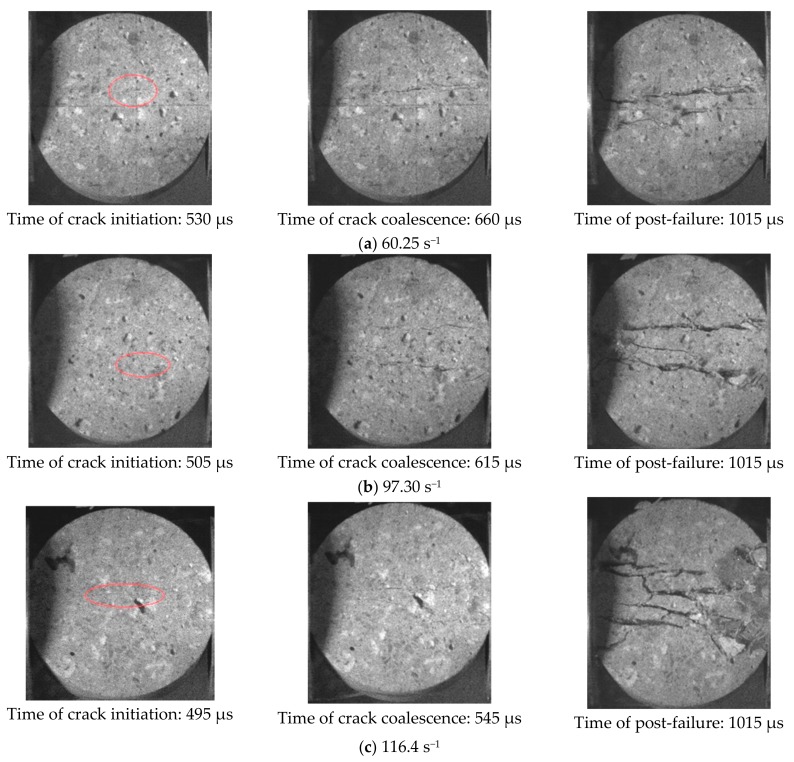
Failure modes of the concrete at different strain rates. (**a**) Strain rate is 60.25 s^−1^; (**b**) strain rate is 97.30 s^−1^; (**c**) strain rate is 116.4 s^−1^.

Figure 7 shows the failure process for concrete subjected to different strain rates. The nine pictures (three for each strain rate) represent crack initiation, crack coalescence, and the final results captured by a high-speed camera, respectively. The moment when the incident wave is transmitted to the strain gauge is regarded as the initial time. In at these three strain rates, specimens crack from the center, which agrees with the results of the Brazilian disc test under quasi-static load. As strain rate increases, crack initiation begins earlier, therefore, the time interval from crack initiation to crack coalescence decreases, and the number of cracks in the center of the specimen increases, eventually resulting in a higher degree of fragmentation and greater kinetic energy of the failed specimen. Feng et al. [26] reached the same conclusion. It has been found that, with increasing strain rate, the fracture energy of cracks forming in the concrete increases, as does the kinetic energy of the failed specimen during the dynamic failure process. The reason for the above phenomena is that the higher the strain rate, the higher the input energy change per unit time. Additionally, the rate of dissipated energy is less than that of input energy, thus causing the untimely failure of the specimen, so that excess energy is stored in the specimen in the form of elastic strain energy and will be released as fracture energy and kinetic energy. Therefore, the higher the strain rate, the greater the number of cracks, and the faster the fragments move about.

From the tests, it was found that the proposed “hysteresis effect of energy release” could effectively explain the test results. However, the testing method could only be tested and verified from the phenomena and not by quantification for more detailed verification. Therefore, verification of the enhancement mechanism of the dynamic strength of concrete requires further study.

## 5. Numerical Tests of the Dynamic Brazilian Disc Test

Due to limitations of the existing testing technologies, energy absorbability of the specimen can only be obtained by calculating the stress wave in the strain gauge. It is difficult to conduct a quantitative analysis of the specimen’s fracture energy, kinetic energy, and other energies. The numerical calculation method can reveal the changes in various energies for the concrete specimens at different moments, providing more effective data for the analysis.

### 5.1. Computational Model and Constitutive Model

**Figure 8:**
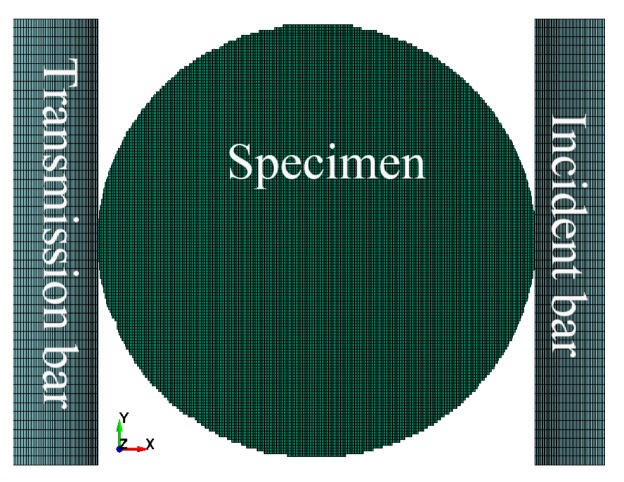
Numerical model of the Brazilian disc specimens.

Ls-dyna finite element software was used to perform the numerical calculations. The computational model was built based on the test mentioned in Section 3.3. The parameters of the experimental bar were adopted as those of the incident bar and the transmission bar. The incident wave was input in the form of a stress wave and loaded on the end surface of the incident bar, away from the specimen. The waveform was obtained based on the results of calculations of the incident wave as measured by tests. Numerical simulation tests were performed under three different gas gun pressures, specifically: 0.15 MPa, 0.20 MPa, and 0.25 MPa. The local grid of the concrete specimen, the incident bar, and the transmission bar are shown in Figure 8.

A linear elastic constitutive model was adopted for the incident bar and the transmission bar. For the concrete specimens, the Holmquist‒Johnson‒Cook (HJC) [35] constitutive model was employed, which can simulate the mechanical properties of materials greatly deformed and at high strain rates. It can also simulate the dynamic response of concrete materials subjected to dynamic loads. As there are 29 material parameters of the HJC constitutive model, and a large amount of research has been mentioned in the literature [36,37], this paper tested the basic parameters of the constitutive model through experiments with other parameters by referring to previous research results. Some of the parameters were adjusted, thus obtaining material parameters applicable to the concrete specimens in this paper, as shown in Table 2.

### 5.2. Results Analysis

#### 5.2.1. Force-Displacement Curves

**Figure 9:**
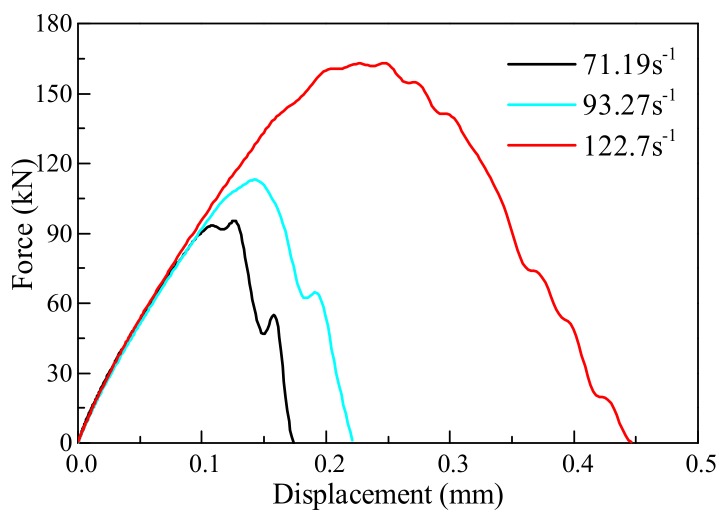
Force-displacement curves of concrete subjected to different strain rates.

Figure 9 shows the force-displacement curves of the dynamic Brazilian disc test at different strain rates. The dynamic tensile strengths of the concrete at strain rates 71.19 s^−1^, 93.27 s^−1^, and 122.7 s^−1^ are 13.22 MPa, 15.65 MPa, and 22.58 MPa, respectively. When the strain rate was increased by 31% from 71.19 s^−1^, the dynamic tensile strength of the concrete was increased by 18.38%; and when the strain rate was increased by 72.34% from 71.19 s^−1^, the dynamic tensile strength of the concrete was increased by 70.8%. This result was similar to the test result in Figure 7. With the strain rate ranging from 60 s^−1^ to 125 s^−1^, the dynamic tensile strength increment of the concrete increased with the strain rate. Thus, the numerical calculation method could simulate the tensile failure of concrete subjected to impact load.

Furthermore, there were some slight differences between the results calculated by the numerical method and the experimental test results. Concrete is a composite material composed of aggregate, mortar and interface. It is not possible to reconstruct the concrete specimen perfectly. The concrete is a homogeneous material in the numerical model, and the crack propagation is not affected by the aggregate. Therefore, the force-displacement curve after the peak force by using the numerical method was more shaken than the experimental test.

#### 5.2.2. Failure Mode

**Figure 10:**
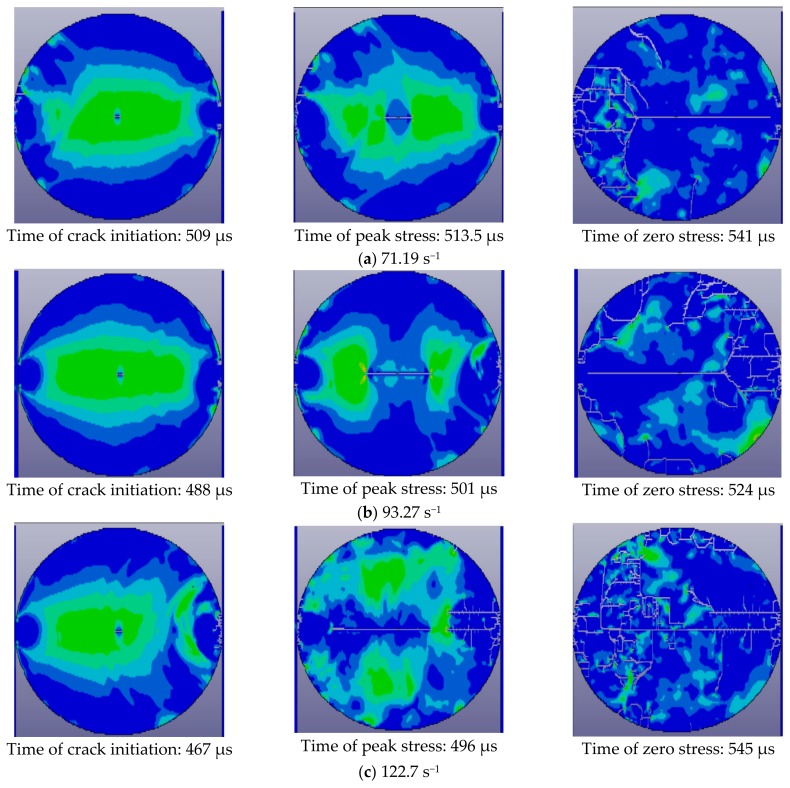
Failure process of the concrete at different strain rates. (**a**) Strain rate is 71.19 s^−1^; (**b**) strain rate is 93.27 s^−1^; (**c**) strain rate is 122.7 s^−1^.

Figure 10 shows the failure process of the concrete at different strain rates. The higher the strain rate, the sooner crack initiation of the concrete begins, the shorter time interval between crack initiation to peak stress of concrete, and the larger the expansion area of the crack. Therefore, the higher the strain rate, the faster the cracks grow. At the moment when the concrete reaches peak stress, the crack area of the concrete increases as strain rate increases. When strain rate is low, the main crack (which develops along the loading direction in the center of the specimen) finally fails. As strain rate increases, secondary cracks in other parts of the specimen gradually grow, leading to a higher degree of fragmentation.

#### 5.2.3. Energy Conversion Process

Before the main crack coalesces, fracture energy is mainly caused by cracking and the expansion of cracks in the specimen. Therefore, in the numerical calculation, the energy of the failed elements in the specimen at different moments before the main crack coalesces can be regarded as the increment of dissipated energy $e_{c}^{\prime }(t)$.

**Figure 11:**
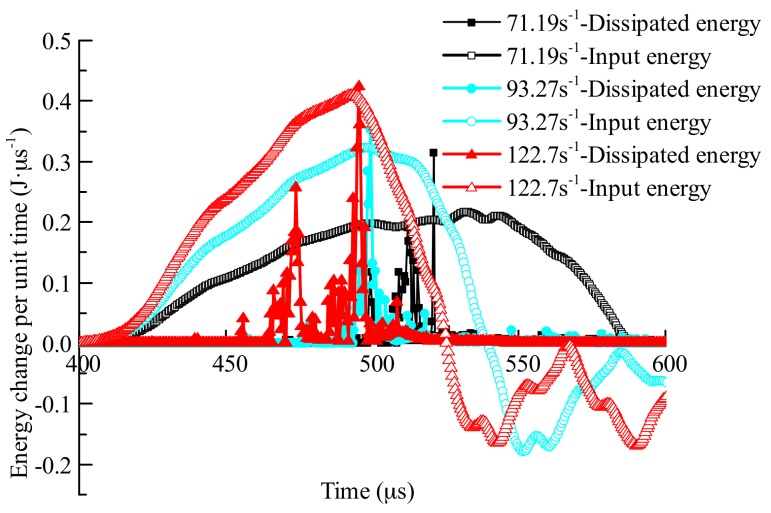
Changes per unit time of input energy and dissipated energy of the concrete specimen.

Figure 11 shows the changes in the processes of input energy and dissipated energy during the concrete failure process. During the dynamic loading process, percent conversion of input energy gradually increases. In the numerical calculation method, initial damage to the concrete is not taken into consideration, therefore there is no fracture generated in the specimen at the initial stage of loading. As loading continues, the center of the specimen begins to crack and the rate of crack propagation gradually increases. When enough elastic strain energy is stored in the specimen, and the percent conversion of dissipated energy is higher than that of input energy, the concrete reaches peak stress. After the peak stress moment, the kinetic energy of elements at both sides of cracks, which are caused by cracking of the concrete, increases by a large margin. Consequently, much elastic strain energy is converted into kinetic energy in the failed part, part of the elastic strain energy is converted into fracture energy, and eventually the concrete fails. Therefore, after the peak stress moment, the energy in failed elements are lower than that of the input energy per unit time.

When the three strain rates were 71.19 s^−1^, 93.27 s^−1^, and 122.7 s^−1^, the moments $e_{e}^{\prime }\left(t\right)=0$ were 514 μs, 500 μs, and 495.5 μs, respectively. The differences between these data and the peak stress moments calculated through stress waves in Table 3 were less than 0.5 μs. These findings prove the accuracy of the analysis on the principle of energy changes. It was found that, as strain rate increased, the rate of change of input energy increased as did the maximum conversion rate of dissipated energy. In addition, the moment when elements began to fail cam earlier, and the intersection point of dissipated energy conversion rate and input energy conversion rate also came earlier, indicating that the moment when concrete reached peak stress occurred sooner.

After the intersection of input energy conversion rate and dissipated energy conversion rate, the energy change of input energy per unit time gradually decreased—and the concrete’s capacity to absorb energy weakened. Furthermore, the higher the strain rate, the sooner the decrease in rate of change of input energy begins. By combining this with the failure process of the concrete in Figure 10, it was found that the decrease in rate of change of input energy per unit time was mainly determined by the degree of failure of the concrete. As strain rate increased, the failure of the part of the specimen which is in contact with the incident bar and transmission bar occurred sooner, so that the stress waves of the incident bar could not be transmitted to the specimen.

## 6. Conclusions

Research into the enhancement mechanism of the dynamic strength of concrete forms the basis for improvement and further enhancement of this dynamic strength. In this paper, the enhancement mechanism of the dynamic strength of concrete was studied from the perspective of energy conversion; and the energy conversion in the failure process of concrete was further studied through dynamic Brazilian disc tests and numerical tests of the dynamic Brazilian disc. The following conclusions were obtained:

(1) Through analysis of the law of energy conversion from the microscopic perspective, it was found that elastic strain energy plays a role in transmitting input energy and dissipated energy in the failure process of concrete—making it a deciding factor in concrete strength. When the rate of the change of elastic strain energy is 0, the concrete reaches peak stress.
(2) By studying the energy conversion law in the failure process of concrete subjected to dynamic load, from the perspective of energy, it was proposed that the dynamic strength of concrete was due to the hysteresis effect of energy release. If the dynamic strength of concrete needs to be further enhanced, future studies from the perspective of delaying facture energy release can be conducted.
(3) Through dynamic Brazilian disc tests, and numerical tests of the dynamic Brazilian disc for concrete, an analysis was carried out into the energy conversion process in the failure process of concrete. The proposed enhancement mechanism of the dynamic strength of concrete was verified by the test results. The moment of the intersection between the rate of change of input energy and the rate of change of dissipated energy was the same as the peak stress moment calculated by stress waves. After the peak stress moment, most of the excess elastic strain energy was converted into kinetic energy of the concrete fragments. Furthermore, the higher the strain rate of loading, the more the elastic strain energy was stored, and the greater the kinetic energy of the fragments.

## Figures and Tables

**Table 1:** Test results at different strain rates.

Test No.	D (mm)	L (mm)	Impacting Pressure (MPa)	*P*_max_ (kN)	Dynamic Tensile Strength (MPa)	Strain Rate (s^−1^)
1	98.18	48.78	0.15	108.32	13.83	51.6
2	98.14	47.65	0.15	110.91	14.50	60.25
3	98.21	49.1	0.15	112.18	14.22	54.3
4	98.20	45.81	0.15	109.33	14.86	73.4
5	98.20	46.8	0.20	119.38	15.88	96.7
6	98.21	45.27	0.20	118.65	16.32	99.7
7	98.24	45.61	0.20	116.64	15.92	97.30
8	98.21	46.46	0.20	115.40	15.46	91.3
9	98.18	48.65	0.25	157.38	20.15	123.8
10	98.20	48.93	0.25	159.41	20.29	114.0
11	98.21	47.12	0.25	160.53	21.21	116.4
12	98.21	49.12	0.25	159.22	20.18	109.3

**Table 2:** Holmquist‒Johnson‒Cook (HJC) model parameters of concrete.

***P* (kg·m^−3^)**	***G* (GPa)**	**A**	**B**	**C**	**N**
2280	14.86	0.79	1.60	0.007	0.61
***f_c_* (MPa)**	***T* (MPa)**	***K*_1_ (GPa)**	***K*_2_ (GPa)**	***K*_3_ (GPa)**	***F_s_***
50	3	85	−171	208	0.004

**Table 3:** Results of numerical tests at different strain rates.

Test No.	D (mm)	L (mm)	Impacting Pressure (MPa)	*P*_max_ (kN)	Time of *P*_max_ (μs)	Dynamic Tensile Strength (MPa)	Strain Rate (s^−1^)
1	98	45	0.15	95.38	513.5	13.22	71.19
2	98	45	0.20	112.935	501	15.65	93.27
3	98	45	0.25	162.96	496	22.58	122.7
